# *Ixeris dentata* (Thunb. Ex Thunb.) Nakai Extract Inhibits Proliferation and Induces Apoptosis in Breast Cancer Cells through Akt/NF-κB Pathways

**DOI:** 10.3390/ijms18020275

**Published:** 2017-01-27

**Authors:** Seong-Ah Shin, Hae-Nim Lee, Gang-Sik Choo, Hyeong-Jin Kim, Jeong-Hwan Che, Ji-Youn Jung

**Affiliations:** 1 Department of Companion and Laboratory Animal Science, Kongju National University, Yesan 32439, Korea; shinsaya@naver.com (S.-A.S.); lhn2726@naver.com (H.-N.L.); dlscjsgkszkf@naver.com (G.-S.C.); tigershout@kongju.ac.kr (H.-J.K.); 2 Biomedical Center for Animal Resource Development, Seoul National University College of Medicine, Seoul 03080, Korea; 3 Biomedical Research Institute, Seoul National University Hospital, Seoul 03080, Korea

**Keywords:** *Ixeris dentata*, breast cancer, apoptosis, Akt, NF-κB

## Abstract

*Ixeris dentata* (Thunb. Ex Thunb.) Nakai (ID) exhibits various physiological activities, and its related plant derived-products are expected to represent promising cancer therapeutic agents. However, the anticancer effects of ID extract on breast cancer cells classified as estrogen receptor (ER), progesterone receptor (PR), and human epidermal growth factor receptor 2 (HER2) are still unknown. In this study, we investigated the anti-cancer effects and analyzed the molecular mechanism of ID extract in T47D, MCF-7 (ER-, PR-positive, HER2-negative), SK-BR-3(ER-, PR-negative, HER2-positive), and MDA-MB-231 (Triple-negative) through in vitro studies. Additionally, we examined its anti-tumor effects through in vivo studies. Our findings indicated that ID extract-induced apoptosis was mediated via various survival pathways on four breast cancer cells by identifying the factors including Bcl-2 family, phospho-Akt and phospho-nuclear factor-κB (NF-κB). Based on in vitro findings that induced apoptosis via Akt-NF-κB signaling, we investigated the effects of ID extract on mice bearing MDA-MB-231 cells. The results showed that ID extract significantly decreased MDA-MB-231 tumor volume and weight via inducing apoptosis by suppressing phospho-Akt. Overall, these results indicate that ID extract induces apoptosis through the Akt-NF-κB signaling pathway in MDA-MB-231 breast cancer cells and tumors, and it may serve as a therapeutic agent for triple-negative human breast cancer.

## 1. Introduction

Breast cancer is the most common type of cancer to affect women worldwide, accounting for 23% of all cancer diagnoses and 14% of cancer-related deaths [1]. Various genetic and environmental factors, including family history and a Westernized diet, are regarded as the major risk factors for breast cancer, but the exact cause has not yet been identified [2]. Breast cancer can be categorized according to the expression of hormone receptors, including estrogen receptor (ER), progesterone receptor (PR), and human epidermal growth factor receptor 2 (HER2). ER-negative breast cancers, which account for almost 25%–30% of all breast cancers, have a poorer prognosis than that of ER-positive cancers [3,4]. However, all types of breast cancer have a risk of relapse, and prognosis is poor after breast cancer recurrence [5,6]. Therefore, an exploration of novel therapeutic agents is needed for the treatment and prevention of breast cancer.

*Ixeris dentata* (ID) is an edible and officinal herb typically used for treatment of indigestion, pneumonia, hepatitis contusion, and tumors in Northeast Asia, including Korea [7]. It contains aliphatics, triterpenoids, cymaroside, and sesquiterpene glycosides [8,9,10], and has various physiological functions, including neuroprotective [11], anti-mutagenic [12], anti-hyperlipidemic [13], anti-inflammatory [14], anti-allergic [15], and anti-proliferative activities [16]. However, the anti-tumor effects of ID extract on human breast cancer cells are unknown.

Akt, also known as protein kinase B (PKB), belongs to the serine/threonine kinase family comprised of PKB family members, including PKBα/Akt1, PKBβ/Akt2, and PKBγ/Akt3 in mammalian cells [17]. Akt, a downstream effector of PI3-kinase, and it plays important roles in signaling pathways in response to growth factors and other extracellular stimuli to modulate several cellular functions, including nutrient metabolism, angiogenesis, and cell migration, growth, apoptosis, and survival [18,19]. In addition, Akt is the major upstream factor activating and regulating nuclear factor-κB (NF-κB) via phosphorylation of p65 by IκB kinase (IKK) both directly and indirectly [20]. Therefore, Akt may confer some of its pro-survival effects by interacting with other pathways and may help increase the efficacy of new therapeutic agents.

Transcription factor NF-κB is a main regulator of the immune response and is involved in the development and progression of diseases such as autoimmune diseases and cancer [21]. The NF-κB family consists of five members: RelA, RelB, c-Rel, NF-κB1 (p105/p50), and NF-κB2 (p100/p52) [22]. Normally, NF-κB dimers (p50/p65) interact with inhibitors of NF-κB (IκBs), IkBα, IkBβ, and IkBε in the cytoplasm. In most cases, activation of NF-κB is dependent on phosphorylation of the IKK complex, which includes IKKα, IKKβ, and IKKγ. Upon phosphorylation by IKK, IκBs are targeted for ubiquitination and proteasomal degradation [23,24]. The activated NF-κB inhibits apoptosis by inducing the expression of anti-apoptosis genes such as Bcl-xL, cellular inhibitor of apoptosis, caspase inhibitors, and c-Myc, and it also induces the expression of a number of target genes involved in cell growth, differentiation, and the inflammatory response [25,26]. Thus, the regulation of NF-κB suggests that it plays a pivotal role in the progression of breast cancer, not only in vitro but also in vivo.

In this study, we compared the anti-cancer efficacy of ID extract in the human breast cancer cell lines T47D, MCF-7, SK-BR-3, and MAD-MB-231 through in vitro studies, and demonstrated anti-tumor effect though in vivo studies by using the breast cancer cell that induced apoptosis significantly. This study highlights the potential medicinal applications of ID extract, a naturally derived product that may serve as a novel therapeutic agent for human breast cancer.

## 2. Results

### 2.1. Effects of Ixeris dentata (ID) Extract on Survival Rate Inhibition in T47D, MCF-7, SK-BR-3, and MDA-MB-231 Cells

**Figure 1:**
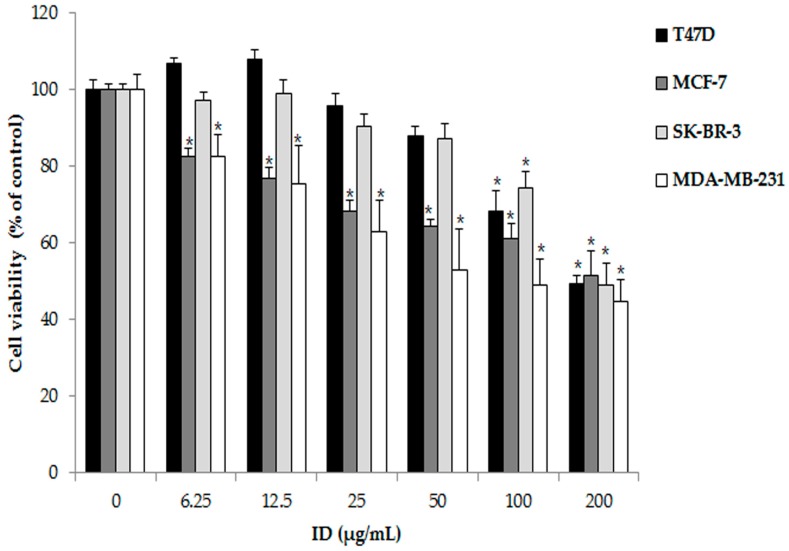
Effects of *Ixeris dentata* (ID) extract on the cell viability in breast cancer cells. T47D, MCF-7, SK-BR-3, or MDA-MB-231 cells were treated with various concentrations of ID extract for 24 h, and cell viability was determined by 3-(4,5-Dimethylthiazol-2-yl)-2,5-diphenyltetrazolium bromide (MTT) assay. Results are presented as means ± standard deviation (SD) of three independent experiments. * *p* < 0.05 was considered to indicate statistical significance compared with non-treated controls.

To identify the effect of ID extract on the survival rate of breast cancer cells, T47D, MCF-7, SK-BR-3, and MDA-MB-231 cells were treated with various concentrations of ID extract (0, 6.25, 12.5, 25, 50, 100, or 200 µg/mL) for 24 h, and the viability of cells was measured as compared with untreated controls using the 3-(4,5-Dimethylthiazol-2-yl)-2,5-diphenyltetrazolium bromide (MTT) assay. As shown in Figure 1, ID extract inhibited cell viability in a dose-dependent manner in MCF-7 and MDA-MB-231 cells, whereas the viability of T47D and SK-BR-3 cells were unaltered at ID extract concentrations ≤50 µg/mL. MDA-MB-231 cells were strongly susceptible to ID extract treatment. Treatment with 100 or 200 µg/mL ID extract for 24 h resulted in a significant decrease in cell viability in the T47D, MCF-7, SK-BR-3, and MDA-MB-231 cells. These results suggest that ID extract induces cell death and inhibits cell viability in T47D, MCF-7, SK-BR-3, and MDA-MB-231 cells at concentrations ≥100 µg/mL.

### 2.2. Effects of ID Extract on Morphological Changes in T47D, MCF-7, SK-BR-3, and MDA-MB-231 Cells

**Figure 2:**
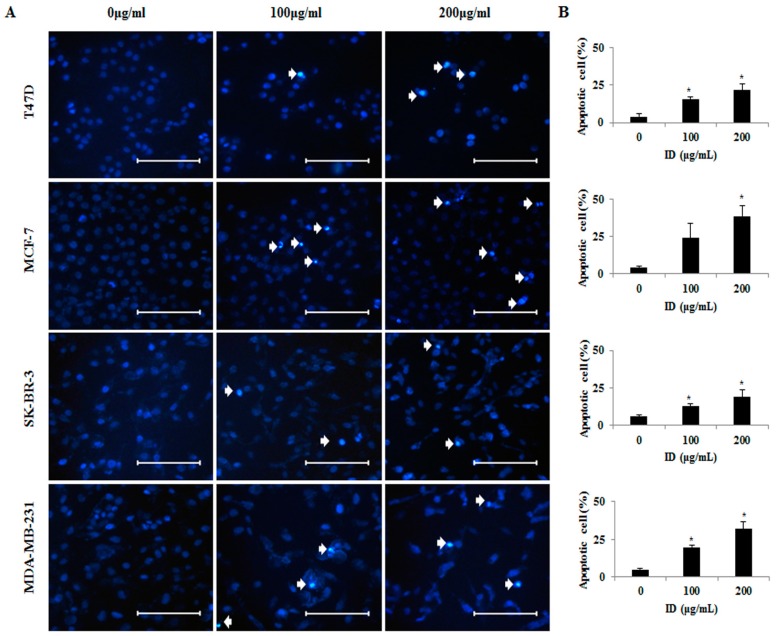
Effects of ID extract on the chromatin condensation in breast cancer cells. (**A**) T47D, MCF-7, SK-BR-3, or MDA-MB-231 cells were treated with ID extract for 24 h. Apoptotic bodies were stained with DAPI (4′,6-diamidino-2-phenylindole). Arrows indicate chromatin condensation. Cleaved nuclei were examined using fluorescence microscopy (200×); (**B**) As shown in the right panel, apoptotic cell numbers of T47D, MCF-7, SK-BR-3, are MDA-MB-231 are presented as percentages. The indicated bar is 10 µm. * *p* < 0.05. ID: *Ixeris dentata*.

DAPI (4′,6-diamidino-2-phenylindole) staining was performed to identify the effect of ID extract on morphological changes associated with the nuclear and chromosomal condensation. T47D, MCF-7, SK-BR-3, and MDA-MB-231 cells were treated with or without 100 or 200 µg/mL ID extract for 24 h. Apoptotic cells after DAPI staining were observed by fluorescence microscopy. As shown in Figure 2A, the DAPI-positive cells were considerably more likely found in cells treated with 100 or 200 µg/mL ID extract as compared with the untreated control group. Moreover, the number of apoptotic cells increased in a dose-dependent manner by ID extract, but apoptotic cells were more often observed in MCF-7 and MDA-MB-231 cells as compared with T47D and SK-BR-3 cells (Figure 2B). These results indicate that ID extract inhibited the cell viability of T47D, MCF-7, SK-BR-3, and MDA-MB-231 cells by inducing apoptosis.

### 2.3. Inhibitory Effects of ID Extract on Akt Kinase Activation

**Figure 3:**
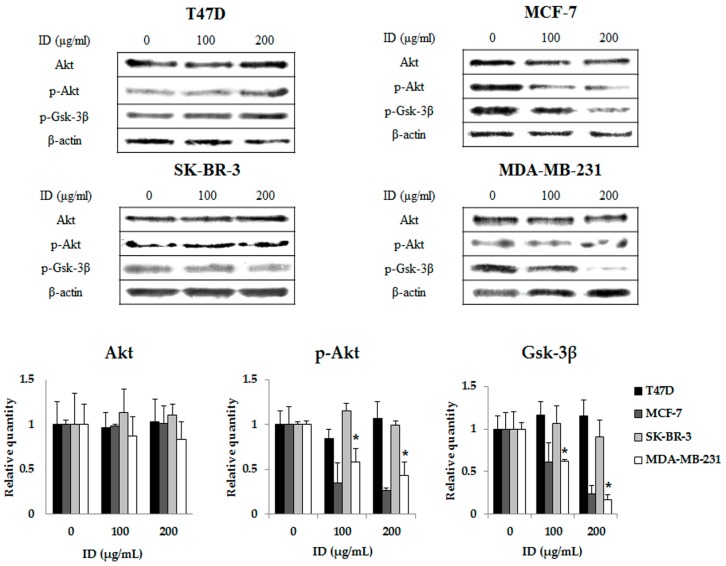
Effects of ID extract on Akt signaling pathway in breast cancer cells. Cells were treated with absence or presence of ID extract for 24 h and harvested to measure protein levels of Akt, p-Akt, and p-GSK-3β (phosphor-glycogen synthase kinase-3β) by western blotting. We used the housekeeping protein β-actin as a positive loading control in all experiments. The bars in the graphs represent the mean ± SD calculated from three independent experiments. * *p* < 0.05. ID: *Ixeris dentata*.

To verify the effect of ID extract on the Akt cell survival pathway, T47D, MCF-7, SK-BR-3, and MDA-MB-231 cells were treated with ID extract for 24 h and analyzed by western blot. The results revealed that phosphorylation (p) of Akt at Ser473 was decreased in a dose-dependent manner in MCF-7 and MDA-MB-231 cells, whereas no changes were observed in T47D or SK-BR-3 cells compared with the non-treatment group. The expression of the Akt downstream effector glycogen synthase kinase-3β (Gsk-3β) was also significantly decreased in MCF-7 and MDA-MB-231 cells, but not in T47D or SK-BR-3 cells (Figure 3). These results indicate that ID extract induced apoptosis through the inhibition of Akt activation in MCF-7 and MDA-MB-231 breast cancer cells.

### 2.4. Inhibitory Effects of ID Extract on NF-κB Kinase Activation

**Figure 4:**
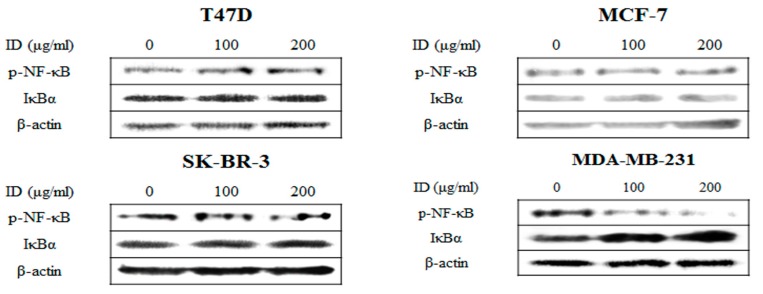

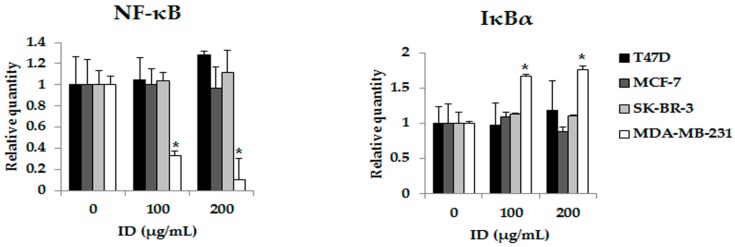
Effects of ID extract on NF-κB signaling pathway in breast cancer cells. Cells were treated with absence or presence of ID extract for 24 h and harvested to measure protein levels of p-NF-κB and IκBα, which were measured by western blotting. We used the housekeeping protein β-actin as a positive loading control in all experiments. The bars in the graphs represent the mean ± SD calculated from three independent experiments. * *p* < 0.05. ID: *Ixeris dentata*.

To confirm whether NF-κB is involved in ID extract-induced apoptosis, we examined the expression levels of NF-κB and its linked protein, IκBα, in ID extract-treated cells. As shown in Figure 4, the expression levels of NF-κB were decreased and the expression levels of IκBα were increased in MDA-MB-231 cells. These results suggest that the inhibition of NF-κB activation may be the underlying mechanism by which ID extract induces apoptosis in ER-negative breast cancer cells.

### 2.5. Effects of ID Extract on the Expression of Apoptosis Pathway Proteins

**Figure 5:**
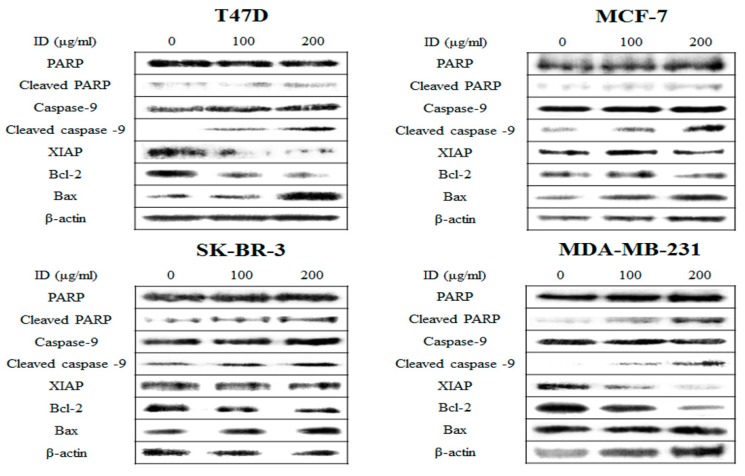

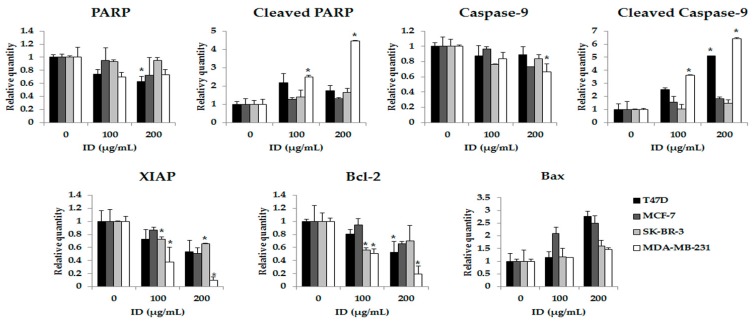
Effects of ID extract on apoptosis-related protein expression in breast cancer cells. Cells were treated with absence or presence of ID extract for 24 h and harvested to measure protein levels of apoptosis pathway by western blotting. We used the housekeeping protein β-actin as a positive loading control in all experiments. The bars in the graphs represent the mean ± SD calculated from three independent experiments. * *p* < 0.05. ID: *Ixeris dentata*.

To evaluate the apoptosis-inducing effect of ID extract in breast cancer cells, T47D, MCF-7, SK-BR-3, and MDA-MB-231 cells were treated with or without 100 or 200 µg/mL ID extract and analyzed by western blot. As shown in Figure 5, the expression of Bax, a pro-apoptotic factor, increased, whereas the expression of Bcl-2, an anti-apoptotic factor, decreased in a concentration-dependent manner. Additionally, we found that expression levels of cleaved caspase-9 and PARP (poly(ADP-ribose)polymerase) increased, whereas expression levels of XIAP (X chromosome-linked inhibitor of apoptosis), an anti-apoptotic factor, decreased following ID extract treatment. These results suggest that the apoptotic mechanism induced by ID extract in breast cancer cells is associated with Bcl-2 family proteins.

### 2.6. Inhibition of Effects of ID Extract on Tumor Growth in Nude Mice

**Figure 6:**
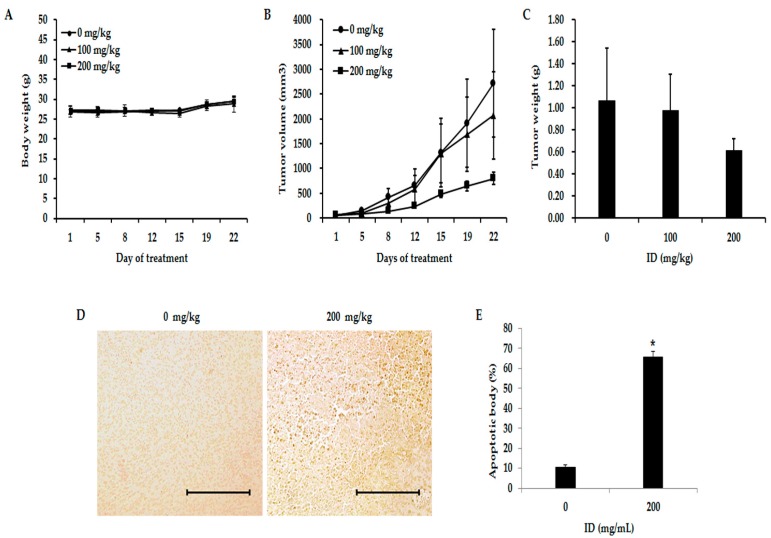
Effects of ID extract on inhibition of MDA-MB-231 breast cancer tumor growth and induction of apoptosis. Mice received an injection of MDA-MB-231 cells and were divided into three groups. ID extract was administered orally five times per week. On day 22, mice were sacrificed and the tumors were excised; (**A**) Body weight curves of MDA-MB-231 cell-bearing mice; (**B**) The tumor volume curves of MDA-MB-231 cell-bearing mice; (**C**) The tumor weight bars of MDA-MB-231-bearing mice; (**D**) Tumor tissues were analyzed by terminal deoxyribonucleotide transferase-mediated dUTP nick end-labeling (TUNEL) assay. Paraffin-embedded tumors were sectioned to a thickness of 5 µm. The slides were examined under a microscope and photographed (200×). Indicated bar is 10 µm; (**E**) Apoptotic body number of MDA-MB-231 tumor presented as percentages. The results are expressed as the mean ± SD. * *p* < 0.05. ID: *Ixeris dentata*.

**Figure 7:**
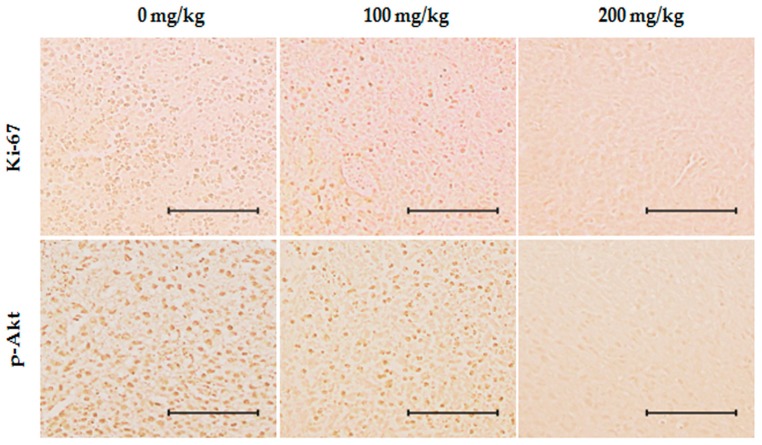
Effects of ID extract on Ki-67 and phospho-Akt (p-Akt) expression. Paraffin-embedded tumors were sectioned to a thickness of 5 µm and analyzed by immunohistochemistry to identify Ki-67 and p-Akt. The slides were examined under a microscope and photographed (400×). Indicated bar is 5 µm. ID: *Ixeris dentata*.

Based on the in vitro findings of the anticancer potential of ID extracts through Akt/NF-κB pathway in breast cancer cells, we examined the in vivo effects of ID extract on breast tumor growth using MDA-MB-231 breast cancer xenograft models. None of the doses had any detectable toxicity reaction, and there were no statistically significant effects on the behavior or appearance of the mice (data not shown). In addition, there was not a significant alteration in body weight (Figure 6A). As shown in Figure 6B, MDA-MB-231 tumor volume significantly reduced in mice treated with 100 or 200 mg/kg ID extract as compared with control (*p* < 0.05). A significant reduction in the MDA-MB-231 tumor size on day 12 was observed in the group treated with ID extract compared with the control. These trends persisted over time, and the reduction was greatest on day 22. On day 22, the respective mice were sacrificed and the tumors resected. Compared with the control, the mean tumor weight was reduced by the ID extract treatment (Figure 6C). As shown in Table 1, the MDA-MB-231 mice treated with ID extract showed a 24% reduction in tumor size in the 100 mg/kg group and a 70% reduction in the 200 mg/kg group on day 22 (both *p* < 0.05 compared with the control group, 0 mg/kg). A terminal deoxyribonucleotide transferase-mediated dUTP nick end-labeling (TUNEL) assay was performed to examine the effect of ID extract on apoptotic cell death in the tumor tissues. As shown in Figure 6D,E, an increase of the number of TUNEL-positive cells was observed in the tumor tissue of MDA-MB-231-bearing mice treated with 200 mg/kg ID extract as compared with the control mice (*p* < 0.05). In addition, immunohistochemistry was performed to investigate the effects of ID extract on expression levels of Ki-67, the protein responsible for the rate of tumor growth, and p-Akt, the protein for confirmation of association with in vitro studies. Ki-67 and p-Akt levels were decreased in a concentration-dependent manner (Figure 7). These findings suggest that ID extract strongly inhibited the growth of breast cancer MDA-MB-231 tumors by inducing apoptosis of tumor cells.

### 2.7. Toxicity Evaluation of ID Extract in Liver and Kidney Tissues

**Figure 8:**
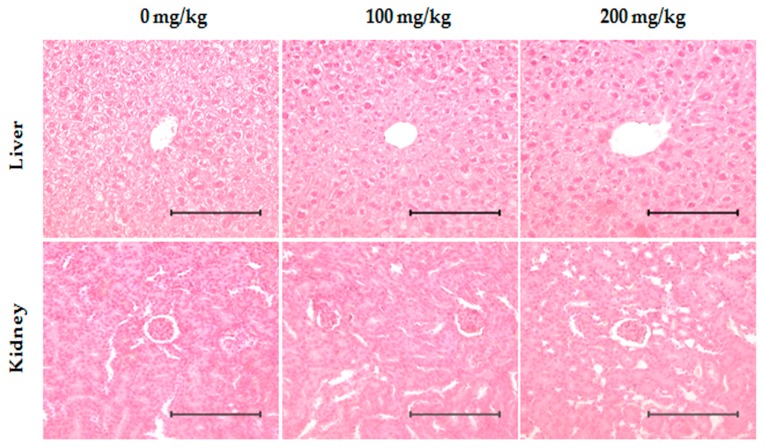
Mice received an injection of MDA-MB-231 cells and ID extract was orally administered five times per week for 22 days. In the end of experiment, mice were sacrificed, and then liver and kidney were excised and evaluated by hematoxylin and eosin (H&E) staining (200×). The dose of ID extract had no detectable toxicological effect on nude mice. Indicated bar is 10 µm.

To ensure the safety of ID extract administration as a therapeutic agent for cancer, mice bearing MDA-MB-231 were sacrificed at the end of the experiment, and liver and kidney tissues were removed. The tissues were fixed in formalin for histopathological evaluation and subjected to hematoxylin and eosin staining. No pathological alterations were observed in the ID extract-treated groups compared with the control group (Figure 8).

## 3. Discussion

Breast cancer is a common neoplasm in women in both developed and developing countries [27]. Breast cancer can be classified according to the expression of ER, PR, and HER2. Although the most common forms of breast cancer are ER positive, approximately one-third of breast cancers are ER negative, which confers a poorer prognosis [28]. Therefore, it is essential to find new effective natural product-derived therapeutics for both ER-positive and ER-negative breast cancers.

In this study, we demonstrated that ID extract possesses anti-cancer effects by inducing apoptosis via p-Akt/NF-κB-dependent downregulation in triple-negative breast cancer cells through comparison of ER-positive and ER-negative breast cancer cells using an in vitro study. In addition, we confirmed that ID extract possesses the development potential as an anticancer agent of ID extract in triple-negative breast cancer tumors using an in vivo study.

To determine the anti-proliferative effect of ID extract on ER-positive and ER-negative breast cancer cells, an MTT assay was conducted. ID extract induced a potent dose-dependent decrease in the number of MCF-7 and MDB-MB-231 cells, whereas it had little effect on the number of T47D and SK-BR-3 cells at concentrations ≤50 µg/mL. However, a significant reduction in cell number was observed at concentrations ≥100 µg/mL in all of breast cancer cells. Overall, these data suggest that ID extract inhibits the growth of human breast cancer cells. In addition, the anti-proliferative effect of ID extract is consistent with the findings of previous studies in lung, liver, colon, and breast cancer cells [15,29]. Therefore, ID extract is believed to be an effective manner to inhibit breast cancer cell proliferation irrespective of ER.

Apoptosis, a form of physiological cell death, is characterized by various forms, including cell shrinkage, chromatin condensation, caspase activation, membrane lipid rearrangement, DNA fragmentation, and cell fragmentation [30]. The induction of apoptosis is one of the most effective approaches to treating cancer. To determine whether the ID extract-induced death of breast cancer cells involved apoptosis, we performed DAPI staining. As shown in Figure 2, ID extract induced potent inhibition of cell viability and substantial chromatin condensation, which indicated that apoptosis was involved. In addition, there was a significant reduction in the number of cells as the ID extract treatment dose increased in all of the cell lines. These results are similar to those of other studies on human breast cancer cells using various natural-derived products, including ID extract [31,32]. Further studies proceeded to elucidate the mechanisms behind the reduction of cell viability and the induction of apoptosis on breast cancer cells using ID extract.

Akt signaling is an important regulator of several cellular functions such as cell growth, apoptosis, and survival. Overexpression of Akt has been found in various human malignancies [18,33]. The Akt signaling pathway also promotes activation of the NF-κB signaling pathway, which inhibits apoptosis [34]. In addition, activity of Akt phosphorylation by growth factor inhibits apoptosis by inducing phosphorylation of Ser 9 in Gsk-3β, a downstream target protein of the Akt signaling pathway [35,36]. Therefore, inhibition of the Akt pathway may be an effective way to prevent and treat malignancies. In the present study, we examined whether ID extract inhibits Akt and Gsk-3β phosphorylation on breast cancer cells. ID extract had no effect on the levels of Akt or Gsk-3β phosphorylation on T47D and SK-BR-3 cells, but it suppressed the levels of Akt and Gsk-3β phosphorylation on MCF-7 and MDA-MB-231 cells. There was a difference between cells, but ID extract-mediated suppression of Akt signaling pathways was identified regardless of ER. These results suggest that inhibition of Akt activation is potentially one of the underlying mechanisms of ID extract-induced apoptosis in breast cancer cells.

NF-κB is involved in multiple cellular functions, including the cell cycle, immune response, and apoptosis. In response to activating stimuli, IKK is activated by the degradation IκBα, a cytoplasmic inhibitor, to allow its activation of NF-κB, and activated NF-κB then promotes cellular resistance to cancer treatments [22,23]. In addition, a correlation between apoptosis and NF-κB has been reported in a number of previous studies [37,38]. In this study, ID extract only inhibited the nuclear translocation of NF-κB by suppressing IκBα degradation in MDA-MB-231 cells, but not in the other cell lines. However, for all breast cancer cells ID extract affected apoptosis-regulated gene products involved in pro-apoptotic (Bax) and anti-apoptotic proteins (Bcl-2 and XIAP), as well as apoptosis target (caspase-9) and DNA repair proteins (PARP), thereby suppressing the cell proliferation and inducing apoptosis.

Our in vitro findings showed that ID extract possessed anticancer effects on both ER-positive and ER-negative breast cancer cells. However, ID extract had differential effects on the four cell types evaluated. T47D and SK-BR-3 cells were affected via an unknown pathway, MCF-7 cells were affected via the Akt pathway, and MDA-MB-231 cells were affected via the Akt-NF-κB pathway. Thus, to determine antitumor effects of ID extract on breast cancer tumors involved in Akt-NF-κB pathway based on in vitro findings, animal experiments were conducted using the MDA-MB-231 cells.

The in vivo anticancer efficacy of ID extract was validated by our experiments in mice bearing MDA-MB-231 tumors. As shown in Table 1 and Figure 6B, the tumor volume of MDA-MB-231-bearing mice treated with ID extract showed a significant reduction compared with the control group. In addition, an increase in DNA fragmentation, a marker of apoptosis, was observed by TUNEL assay in MDA-MB-231 tumor tissues from nude mice administered ID extract (Figure 6D,E). These results suggest that the inhibition effect on the tumor correlated with the increased apoptosis level in the tumor cells of the ID extract-treated group.

A Ki-67 was used to differentiate between nuclei of proliferating cells and resting cells, and it is an important factor for the treatment of breast cancer, as previously described [39,40]. In addition, Akt is an important regulator of several cellular functions such as cell growth, apoptosis, and survival, and it is overexpressed in various carcinomas. An immunohistochemical assay confirmed the expression of Ki-67 (proliferation maker) and Akt protein levels, and demonstrated that its expression was decreased in the ID extract-treated mice (Figure 7). In addition, reduction of Akt expression in a concentration dependent manner in the ID extract-treated group corresponds with the in vitro results. Thus, the in vivo findings support our in vitro results and suggest that ID extract inhibits tumor growth by inducing apoptosis in MDA-MB-231 breast tumor cells.

When in the process of developing novel anticancer drugs, the potential toxic effects toward healthy tissues is an important factor to consider in order to prevent or mitigate the side-effects on non-targeted cells [41]. With this in mind, no significant histopathological features were found in the treated with ID extract group as compared with the control group, as supported by the result of a toxicity confirmation on the liver and kidney of the mice treated with ID extract using H&E stain (Figure 8).

## 4. Materials and Methods

### 4.1. Chemicals, Drugs, and Antibodies

3-(4,5-Dimethylthiazol-2-yl)-2,5-diphenyltetrazolium bromide (MTT), Dimethyl sulfoxide (DMSO) were purchased from Sigma-Aldrich (St. Louis, MO, USA). Robwell Park Memoril Institute (RPMI)-1640 medium, penicillin-streptomycin, trypsin-ehylenediaminetetraacetic acid (EDTA), and fetal bovine serum (FBS) were obtained from HyClone Laboratories (Logan, UT, USA). Cell lysis buffer were obtained from Invitrogen (Carlsbad, CA, USA). 4′,6-diamidino-2-phenylindole (DAPI) was purchased Life Technologies (Grand Island, NY, USA). Anti-β-actin (#4967), anti-Bax (#2772), anti-Bcl-2 (#2876), anti-caspase-9 (Human Specific) (#9502), anti-Poly(ADP-ribose)polymerase (PARP, #9542), anti-X chromosome-linked inhibitor of apoptosis (XIAP, #2042), anti-phospho-Akt (Ser473) (D9E) XP^®^ Rabbit (#4060), anti-Akt (#9272), anti-phospho-Glycogen synthase kinase-3β (Gsk-3β) (Ser9) (#9336), anti-phospho-NF-κB p65 (Ser536) (93H1) Rabbit (#3033), anti-IκBα (#9242), anti-Ki-67 (D2H10) Rabbit (IHC Specific) (#9027), and anti-rabbit horseradish peroxidase (HRP)-linked (#7074) antibodies were purchased from Cell Signaling Technology (Beverly, MA, USA). The DeadEnd™ fluorometric terminal deoxyribonucleotide transferase-mediated dUTP nick end-labeling (TUNEL) Assay Kit was purchased from Biovision (San Francisco, CA, USA).

### 4.2. Preparation of Ixeris dentata (Thunb. Ex Thunb.) Nakai (ID) Extract

ID (Voucher specimen no. KRIB 0000226) were purchased from a Korean plant extract bank at the Korea Research Institute of Bioscience & Biotechnology (Cheongju, Korea). The dried ID was powdered in a blender, and exhaustively extracted with MeOH at 45 °C with a sonication for three days. The sonication was performed 15 min, 10 times per day, and extracts were then filtered using nonfluorescent cotton filters (0.45 µm pore size). After concentration to yield the methanol extracts in decompression, the extracts were lyophilized for 24 h and stored at −4 °C. ID extract yield was 13.50%.

ID extract was dissolved in DMSO (0.5%), PBS (mg/mL), and stored at −20 °C. The same amount of DMSO used in the high concentration group was used in each control and experimental group.

### 4.3. Cell Line and Culture

The human breast cancer cell lines T47D, MCF-7 (ER-, PR-positive, HER2-negative), and SK-BR-3 (ER-, PR-negative, HER2-positive), MDA-MB-231 (Triple-negative) were obtained from the Korean Cell Line Band (KCLB, Korea). Breast cancer cells maintained in RPMI-1640 supplemented with 5% FBS and penicillin-EDTA under standard culture conditions at 37 °C with 95% humidified air and 5% CO_2_. The culture medium was renewed every two to three days. For ID extract treatment, breast cancer cells were seeded at a density of approximately 80%–90% in a 175 cm^2^ flask (Nunc, Fisher Scientific, Loughborough, UK) and allowed to attach overnight.

### 4.4. Cell Viability Assay

Cell survival rate was measured by the MTT assay. T47D, MCF-7, SK-BR-3, and MDA-MB-231 cells were seeded in 96-well plates at a density 2 × 10^4^ cells/mL in a volume of 200 µL/well, and incubated for 24 h. After which, cells were treated with 0, 6.25, 12.5, 25, 50, 100, or 200 µg/mL ID extract for 24 h in triplicate independent experiments. The medium was removed, and then the cells were incubated for 2 h with 40 µL (5 mg/mL) of MTT solution per well plates, respectively. The medium was then aspirated, and the formazan product generated by viable cells was solubilized with the addition of 100 µL DMSO. The absorbance of the solutions at 595 nm was determined using a microplate reader (Bio-Rad, Hercules, CA, USA). The percentage of viable cells relative to untreated (control) cells was estimated.

### 4.5. Nuclear Morphology

In order to identify ID extract-induced apoptotic cell death, the T47D, MCF-7, SK-BR-3, and MDA-MB-231 cells were seeded in 60 mm plates at 1 × 10^5^ cells/well, and then incubated with 0, 100, and 200 µg/mL ID extract for 24 h. Following treatment, the cells were fixed in phosphate-buffered saline (PBS) containing 4% paraformaldehyde for 15 min at room temperature, and stained with DAPI. The cells were washed twice with PBS and examined under a fluorescence microscope (IX71, Olympus Co., Tokyo, Japan) at a 200× magnification.

### 4.6. Western Blot Analysis

Cells were cultured in 175 cm^2^ flasks under the same conditions as described above and treated with/without 100 or 200 µg/mL ID extract for 24 h. Then cells were washed twice with PBS and treated with trypsin-EDTA for 1 min. Cell pellets were harvested by centrifugation, lysed in lysis buffer (Invitrogen Life Technologies), and centrifuged at 13,000 rpm for 5 min at 4 °C. Protein samples were stored at −80 °C. Protein concentration was measured using the Bradford protein assay (Bio-Rad). Protein extracts (45 µg) were resolved by sodium dodecyl sulphate-polyacrylamide gel electrophoresis (SDS-PAGE) and transferred into nitrocellulose membranes (Amersham Biosciences, Uppsala, Sweden) by electrophoresis. The membranes were blocked with Tris-buffered saline (TBS) which contained 5% non-fat milk powder and 0.1% Tween^®^ 20 at 4 °C for 1 h 30 min. Next, each of the membranes were incubated with appropriate primary antibodies overnight at 4 °C with gentle shaking and washed with a TBS containing 0.1% Tween^®^ 20 (TBS-T) three times for 10 min. Subsequently, the membranes were incubated with secondary horseradish peroxidase (HRP) conjugated anti-rabbit IgG for 2 h. After washing, the bands were detected using enhanced chemiluminescence (ECL) western blotting detection reagents (Thermo Scientific, Rockford, IL, USA) according to the manufacturer’s instructions. β-Actin was used as a loading control.

### 4.7. Animal and Xenograft

Five-week-old male BLAB/c-nude mice (nu/nu) were purchased from the animal production company of Orient-Bio (Gyeonggi-do, Korea). Animals were maintained at 23 ± 5 °C at 40% ± 10% relative humidity with artificial lighting from 8:00 a.m. to 8:00 p.m. in facilities approved by the Companion and Laboratory Animal Science Department of Kong-Ju National University. Animals were housed in cages and allowed access to laboratory pelleted food (Biopia, Korea) and water ad libitum. All animal experiments were performed with the approval of the Institutional Animal Care and Use Committee following the guidelines of Kong-Ju National University (KNU_2016-03).

MDA-MB-231 cells were injected subcutaneously (1 × 10^7^/mL) into the flank using a 27-gauge needle. When tumors were stabilized, MDA-MB-231 tumor xenograft mice were assigned randomly to each of the three groups with five mice per group. ID extract was orally administered five times per week at a dose of 100 or 200 mg/kg body weight, while vehicle-treated mice were administered orally the PBS containing DMSO 0.5%. Mice were euthanized 22 days after administration. Mice weight and tumor volume were surveyed twice per week. The volumes of tumors were measured using vernier calipers (Mitutoyo, Kawasaki, Japan). After the experiment was over, mice were sacrificed and tumors were excised to measure tumor weight. A portion of the tumor was embedded in paraffin and used for TUNEL assays and immunohistochemistry (IHC).

Volume (mm^3^) = 0.5 × length × width^2^

### 4.8. TUNEL Assay

Apoptotic cell death was observed using a Biovision DeadEnd™ fluorometric TUNEL system kit according to the manufacturer’s instructions. Briefly, tumor tissues were fixed in 10% formalin overnight and embedded in paraffin. Blocks were then cut into 5-µm-thick slices. The sections were attached to microscope slides and were deparaffinized by immersion in xylene. Afterwards they were then washed with 100% ethanol and the samples were rehydrated by sequential immersion in a graded ethanol series (90%, 80%, and 70%). Tumor sections were visualized using 3′-diaminobenzidine tetrahy-drochloride (DAB) solution, treated with mounting reagent, and observed under a microscope (200×).

### 4.9. Immunohistochemistry

Tumor sections were deparaffinized with xylene twice for 10 min, and rehydrated with ethanol (100% and 90%) for 1 min, and rinsed with tap water for 10 min. Sections were then incubated at 4 °C with anti-phospho-Akt, anti-Ki-67 antibodies overnight and incubated for 1 h at room temperature with a peroxidase-conjugated goat anti-rabbit antibody followed by washing. Tumor sections were visualized using DAB solution, treated with mounting reagent, and observed under a microscope (400×).

### 4.10. Histological Examination

The excised livers and kidneys were immediately fixed in 10% neutral-buffered formalin, and embedded in paraffin. Next, paraffin blocks were cut into 5-µm-thick sections. The sections were examined under a light microscope (200×) following hematoxylin and eosin (H&E) staining.

### 4.11. Statistical Analysis

The Results are presented as the mean ± standard deviations (SD). Differences between mean values for control and ID extract-treated groups were assessed by one-way analysis of variance (ANOVA) with Dunnett’s *t*-tests. *p* < 0.05 was considered to indicate a statistically significant difference.

## 5. Conclusions

ID extract inhibits the growth of T47D, MCF-7, SK-BR-3, and MDA-MB-231 breast cancer cells through the induction of apoptosis regardless of ER. Among the breast cancer cells, MDA-MB-231 triple-negative breast cancer cells displayed the anticancer and apoptosis induction effects via inhibition of Akt phosphorylation and NF-κB binding by ID extract, as observed by in vitro studies. In addition, apoptosis occurred through the inhibition of Akt signaling by ID extract, as observed by in vivo studies. Overall, our results support that ID extract may be useful as an alternative therapeutic drug for the treatment of breast cancer, particularly for patients with triple-negative breast cancer.

## Figures and Tables

**Table 1:** Tumor inhibition rate of mice implanted with MDA-MB-231 breast cancer cells treated with ID extract.

Cell Line	Group	Pre-Experiment	Post-Experiment	Inhibition Rate ^b^ (%)
		Size (mm^3^)	Size (mm^3^)	
MDA-MB-231	0 mg/kg ^a^	53	2719	
	100 mg/kg	61	2076	24
	200 mg/kg	65	801	71

^a^ Control; ^b^ Date are expressed as percent relative to the control. ID: *Ixeris dentata*.

## Abbreviations

DAB	3′-Diaminobenzidine tetrahydrochloride
DAPI	4′,6-Diamidino-2-phenylindole
ECL	Enhanced chemiluminescence
ER	Estrogen receptor
Gsk-3β	Glycogen synthase kinase-3β
HER2	Human epidermal growth factor receptor 2
HRP	Horseradish peroxidase
ID	*Ixeris dentata* (Thunb. Ex Thunb.) Nakai
IKK	IκB kinase
IκBs	Inhibitors of NF-κB
NF-κB	Nuclear factor-κB
PARP	Poly(ADP-ribose)polymerase
PR	Progesterone receptor
TBS	Tris-buffered saline
XIAP	X chromosome-linked inhibitor of apoptosis
